# Impact of primary headaches on subjective sleep parameters among adolescents

**DOI:** 10.4103/0972-2327.42936

**Published:** 2008

**Authors:** Ravi Gupta, Manjeet Singh Bhatia, Devendra Dahiya, Sameer Sharma, Rahul Sapra, Kapil Semalti, Raman Preet Singh Dua

**Affiliations:** Department of Psychiatry, University College of Medical Sciences and GTB Hospital, Dilshad Garden, Delhi, India

**Keywords:** Migraine, sleep, sleep-disruption, tension type headache

## Abstract

**Context:**

Headache patients commonly report sleep disruption and sleep disorders. Available literature suggests that the sleep pattern of headache sufferers is different from the control group. Patients in these studies were recruited from headache clinics; they did not include tension type headache.

**Aims:**

The aim of this study is to find out whether primary headaches affect sleep patterns.

**Settings and Design:**

Community based cross sectional study

**Materials and Methods:**

This study was conducted in three high schools. Children in the 12-19 age group were allowed to participate. They were given a questionnaire in the presence of at least one of the authors, who assisted them in filling it. They were asked to provide responses based on most severe recurrent headache that they had experienced rather than the more frequent ones. The questionnaire included questions regarding demographic data and the characteristics of headache according to International Classification of Headache Disorders-2 criteria. Part B of the questionnaire contained questions regarding sleep habits. The children were asked to provide data regarding sleep habits on a normal school day. Diagnosis was based upon the information contained in the questionnaire. A telephonic interview was also done, where the information provided was found inadequate.

**Statistical Analysis Used:**

Analysis was done with the help of SPSS v. 11.0., descriptive analysis, Chi square, and one way ANOVA with post hoc analysis. Kruskall-Wallis tests were run.

**Results:**

A total of 1862 subjects were included in the study. Migraineurs and tension type headache sufferers comprised 35.7% and 13.4% of the group respectively. Migraineurs had the highest prevalence of nocturnal awakenings (*P* < 0.001), abnormal movements (*P*=0.001) and breathing problems during sleep (*P* < 0.001). Approximately half the migraineurs felt sleepy during the day (*P*< 0.001) and spent around 1.17 hours in sleep during the day (*P* = 0.007). Similarly, values for frequency of nocturnal awakenings per week (*P* < 0.001), wake time after sleep onset and offset (*P* < 0.001 and 0.002 respectively) were the maximum in migraineurs. Only 32.8% migraineurs reported refreshing sleep (*P*< 0.001). Post hoc analysis revealed that migraineurs were different from the other two groups on most of the parameters.

**Conclusions:**

Sleep disruption is more common in migraineurs than those in the tension type headache sufferers and the control group.

## Introduction

The association of headache and sleep is two-way: on the one hand, insomnia and many other sleep disorders, especially obstructive sleep apnea, may cause headache;[1] and, on the other hand, headache significantly impairs the onset of sleep or awakens the persons from sleep.[2]

When we talk about primary headaches, migraine is considered to be intimately related to the sleep. Migraine may occur during sleep; or sleep deprivation can induce migraine. It is also a relieving factor for the headache. Available literature suggests that migraineurs suffer from varied sleep problems, including trouble in falling or staying asleep and alteration in the total duration of sleep. Sometimes migraineurs wake up in the morning with a headache.[2] Other studies conducted among children found that migraine and tension type headache (TTH) sufferers were worse than the control group on the measures of total duration of sleep, sleep latency, abnormal movements during sleep, sleep-quality, frequent awakenings, breathing difficulty during sleep, stage four sleep disorders and daytime sleepiness.[3,4] Besides, studies have reported that children with migraine have higher frequency of snoring, parasomnias and daytime sleepiness than children in the control group as well as those suffering from non-migraine headache.[5] One study even reported that polysomnography changed the diagnosis of almost half the migraine and TTH sufferers to obstructive sleep apnea, periodic limb movement disorder and fibromyalgia.[6]

Many of these studies included only migraineurs and, at least to our knowledge, sleep has never been studied in TTH subjects. In addition, most of the studies did not analyze subjective sleep parameters in detail. Hence, the present study was planned to assess the subjective sleep parameters of primary headache subjects, (migraineurs and TTH subjects) and children without headache, and compare them with each other.

## Materials and Methods

All students of grades ninth to twelfth of three schools were included in the study. Their school health records were screened for the presence of any chronic medical illness that may cause secondary headache e.g., epilepsy and other neurological illnesses, recent trauma, sinusitis, dental problems, uncorrected refraction errors, respiratory illnesses etc. This information was corroborated with the information acquired from the students and when there was an agreement between the two, such cases were excluded from the study. Similarly, students who did not have at least three headache episodes during the past one year or who had three or more headache episodes but did not remember its character were instructed not to fill the questionnaire and were also excluded from the study, thus excluding the recall bias.

The rest of the students were included in the study. The purpose of the study was explained to them by the authors, in the presence of the school physician and the school authorities. They were encouraged to participate and provide reliable information. Their verbal consent was obtained.

### Procedure

This study was based on a questionnaire which had two parts. Part A contained questions based on ICHD-2 diagnostic criteria[7] for various primary headaches and Part B contained questions regarding their sleep habits and questions concerning demographic data.

Part A included questions pertaining to issues such as duration of the headache and change in its severity, frequency and duration since onset, and age of onset of recurrent headache. At the outset, the participants were instructed not to fill the characteristics of the headache that they experience during cold, dental pain, fever etc. If any student suffered more than one type of headache, he was instructed to base his responses relating to the one which was most disabling and recurring. The questionnaire has not been validated previously in a population but it has proven effective for the diagnosis of headache and their categorization according to ICHD-2, in a headache-clinic.

Before giving the children Part B, they were instructed to provide details of the sleep habits that they followed most of the time on school days/nights (excluding holidays and weekends), in the last six months. It included questions such as total duration of sleep in a day, time to bed, sleep latency, nocturnal awakenings (weekly and daily frequency, duration, reasons) etc.

For the present study, sleep parameters were considered pathological when:

- Nocturnal awakening was associated with pain, trouble falling asleep when they occurred more than two times a night and when they recurred more than two days in a week;
- Nonrefreshing sleep occurred more than two days in a week;
- Daytime sleepiness occurred despite adequate night time sleep or when the subjects felt so sleepy during the day that it interfered with their functioning.

While being questioned about the family history of sleep problems, the children were asked to base their answers on the above information.

The questionnaire was distributed to 2563 students in their classrooms and at least one of the authors read it aloud in front of them. Each item of the questionnaire was explained sequentially and students were asked to mark/write the response that applied to them in the response sheet. Students' queries, if any, were resolved immediately. Diagnosis of all the cases were made later and it was based on the ICHD-2 criteria, depending upon the responses provided in the response sheets. Wherever the information provided could not lead to a single diagnosis, the students were contacted telephonically and further information was gathered to facilitate reaching a conclusion. In the present study, we included migraineurs, TTH subjects and controls.

## Results

**Figure 1:**
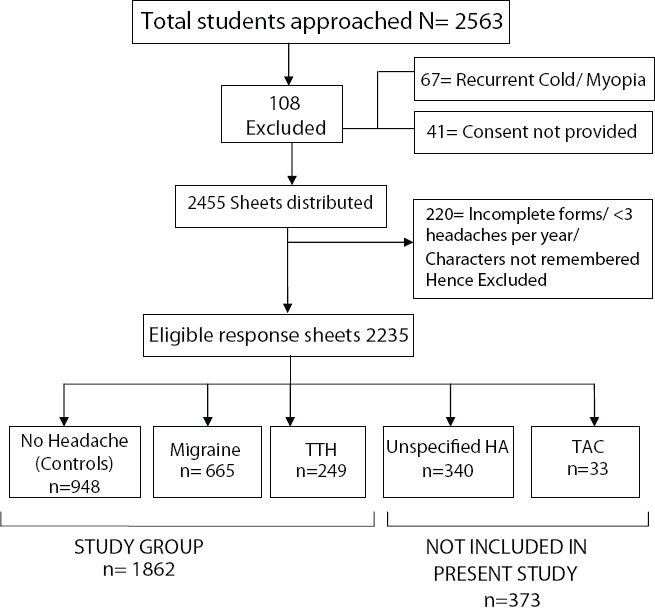
Diagram showing constitution of the sample of the study

**Figure 2:**
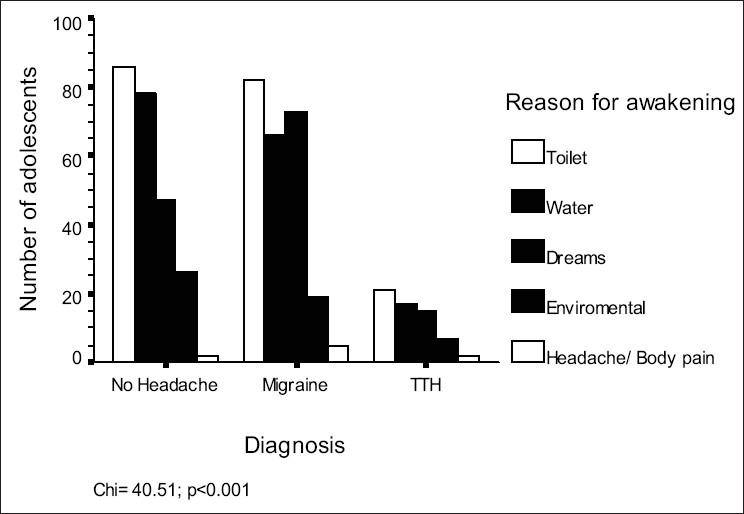
Reasons for awakenings in different headache groups

**Figure 3:**
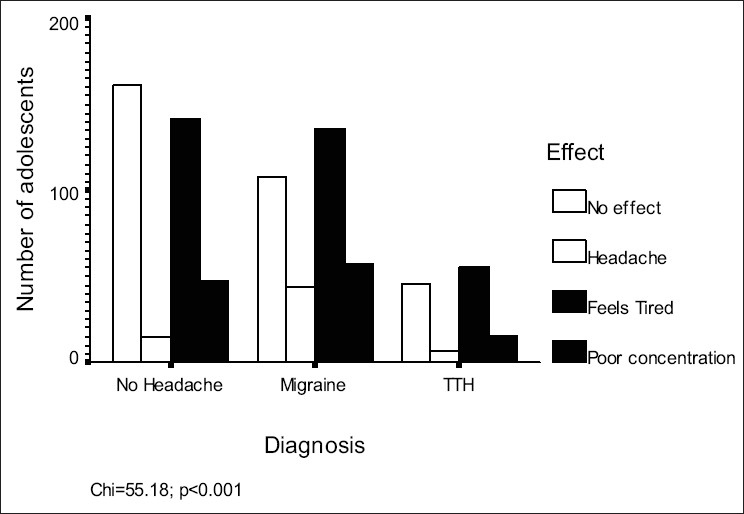
Effects of avoiding daytime nap in sample

In this study, a total of 1862 subjects were included [Figure 1]. Composition of the sample is depicted in Table 1. Time to bed and wake up time were not significantly different among the groups (*P*=0.71 and 0.08 respectively). Prevalence of nocturnal awakening was the highest in the migraine group (42.4%), followed by TTH (31.6%) and controls (29.6%) (*P*<0.001). It must be noted that nocturnal headache caused awakening in a small number of subjects only [Figure 2]. Early morning headache was reported by 2.8% of the sample and its frequency was not different among three groups. Approximately half the subjects in the migraine group felt sleepy during the day; this was not seen in TTH sufferers (*P*<0.001). Both migraine and TTH groups had significantly higher frequencies of daytime naps than the control group (*P*<0.001). Figure 3 shows the effect of avoiding daytime naps. Most of the migraineurs and TTH subjects complained that their sleep decreased during examinations, as compared to those in the control group (p< 0.001). Table 2 depicts the continuous variables among these groups and their post hoc analysis shows that the migraine group was different from the remaining two groups on most of the parameters [Table 3].

**Table 1:** Composition of the study sample (N=1862)

Variable	Frequency
Age (years)	
• Male	15.22 ± 1.25
• Female	15.07 ± 1.20
Gender (%)	
• Male	61.4
• Female	38.6
Headache (%)	
• No Headache	50.9
• Migraine	35.7
• Migraine without aura	16.9
• Migraine with aura	3.8
• Migraine with and without aura	15
• Tension type headache	13.4
• Episodic infrequent TTH	9.1
• Episodic frequent TTH	4.0
• Chronic TTH	0.3

**Table 2:** Comparison of sleep characters in the sample

Variable	Migraine (n=665)	TTH (n=249)	Controls (n=948)	Sig.
Total sleep time (hrs)*	7.96	8.1	7.99	0.43
Sleep latency (min)#	27.85	24.96	64.95	0.671
Nocturnal awakening since…years*	0.56	0.37	0.31	0.63
Frequency of nocturnal awakening / week*	1.40	0.9	0.74	<0.001
Wakes after.. hours*	1.23	0.93	0.83	<0.001
Wake after sleep onset (WASO) (min)*	7.63	4.67	4.48	<0.001
Wake after sleep offset (WASF) (min)*	10.51	8.16	9.03	0.002
Sleep efficiency*	90.58	91.59	92.47	<0.001
Refreshing sleep (%)	32.8	37.3	55.3	<0.001
Duration of daytime sleep (hrs)#	1.17	1.04	0.96	0.007
Family history of sleep problem (%)	18	7.6	11.6	<0.001

* Brown Forsythe Test

# F test

**Table 3:** Post-hoc comparison of sub-groups in the sample

Dependent Variable	Diagnosis	Diagnosis	Sig.	99% C I	
				
				Lower Bound	Upper Bound
Frequency of awakenings per week*	Migraine	Control	0.000	0.34	0.97
		TTH	0.004	0.04	0.94
Wakes after (hours)*	Migraine	Control	0.000	0.12	0.67
		TTH	0.035	-0.05	0.66
WASO (min)*	Migraine	Control	0.000	0.86	5.45
		TTH	0.008	0.07	5.84
WASF (min)*	Migraine	Control	0.015	-0.06	3.02
		TTH	0.000	0.53	4.15
Sleep efficiency*	Migraine	Control	0.000	-3.26	-0.53
		TTH	0.373	-3.21	1.19
Duration of day time sleep (hours)#	Migraine	Control	0.004	0.16	0.38
		TTH	0.391	-0.15	0.39
Frequency of sleep problem/ week*	Migraine	Control	0.000	0.07	0.20
		TTH	0.000	0.03	0.22
Frequency of sleep problems/ night*	Migraine	Control	0.000	0.15	0.49
		TTH	0.000	0.08	0.50

Alpha=0.01

* Games-Howell

# Tukey HSD

Only significant values are shown.

## Discussion

This study shows that primary headache sufferers are different from the control group on most of the sleep parameters. It was most conspicuous in migraineurs, followed by subjects with TTH. The migraineurs had higher chances of fragmented sleep due to frequent awakening, longer time spent waking in the bed and higher frequency of abnormal movements. Similarly, they had higher frequency of nonrefreshing sleep, as suggested by direct questioning as well as longer wake time after sleep offset (WASF), causing sleepiness during the day, higher frequency of daytime napping and longer time spent in naps. Moreover, family history of sleep problems was more common in them. On the other hand, the TTH group was closer to the control group on most of the variables. It is more interesting to note that time to bed, wake-up time, total sleep time, sleep latency and duration since the subjects were suffering from nocturnal awakenings were similar in all the groups, thus suggesting that the sample was homogenous.

Distribution of the primary headache in this sample was in close approximation to the previous proxy-questionnaire based study conducted in children,[3] but in a smaller sample. Time to bed, total sleep time and wake up time were also not different between migraineurs and TTH in that study. However, in that study a significant difference was observed between headache groups and the control group on measures of total sleep time, sleep latency, nocturnal awakenings, and sleep quality. They converted continuous variables into ordinal variables, perhaps to ease the respondent, and it could be one reason why some of their results do not match the present findings. Another possibility for the observed differences could be the inclusion of younger children and proxy responses, since information was provided by parents. Similar to the present findings, the earlier study also reported that migraineurs had higher prevalence of frightening dreams etc., as compared to TTH subjects and those in the control group. Since most of these problems tend to wake up the co-sleeper, accurate information about them can be generated. Perhaps this was the reason behind the good agreement of data between two studies on these parameters.

An important finding in the present study was the reason for the nocturnal awakening among different groups. It must be noted that awakening due to headache or body pain or leg pain was most common in migraineurs, so were the frightening dreams. Similar results in the past have been described and these symptoms were found to be more prevalent among migraine and TTH group as compared to controls,[2,3] and migraineurs in relation to the other two groups respectively.[3]

Sleep problems are not limited to the adolescent migraineurs, as they are also seen in the adult population.[2] A clinic based cohort study reported that migraine subjects complained of trouble falling asleep and difficulty in staying asleep. It was more common in chronic migraineurs, as compared to episodic migraineurs. Furthermore, total sleep time was inversely proportional to the frequency and severity of the headache.[2] About seventy percent of the subjects in that study complained of headache upon waking,[2] whereas it was reported by only three percent of subjects in the present study. It must be noted that early morning headache is an important indicator of headache being an epiphenomenon masking underlying sleep disorder.[6] Therefore, the chances of headache being secondary to sleep-related pathology are very less in this study's sample.

To our knowledge, there are only a few studies that had examined sleep in the tension type headache subjects.[3,5] It had been demonstrated that TTH subjects had higher chances of difficulty in getting sleep; the quality of their sleep was poor; they experienced frequent awakenings and there were more instances of falling asleep in the school, as compared to the control group. Similar results were also seen in the present study. The results were statistically significant, but clinically insignificant, due to the lesser magnitude of difference from the controls. Hence, it supports the results of Isik *et al.*[5] who reported more sleep disturbances in migraineurs as compared to those in the control as well as non-migraine headache groups.

Looking at these findings, it can be assumed that migraine and sleep disorder could have common neurobiological underpinnings. It also suggests that TTH may have different pathogenesis as compared to migraine. It seems plausible, as serotonin,[3,8] melatonin,[9,10] hypothalamus[11] and nitric oxide[12] etc. have been implicated in the pathophysiological regulation of headache as well as sleep and its disorders, but the involvement of these structures was never demonstrated in tension type headache patients. In the past, few evidences have been presented suggesting the pathophysiological overlapping between migraine and TTH,[13–15] but they do not provide adequate explanation of the all symptoms of headaches, except that of biochemical changes noticed during headache episodes. Thus, it is evident from the present study that probably migraine and TTH are two separate diagnostic and pathophysiological entities, and that yet unknown supra-bulbar mechanisms are more important in the generation of migraine. The results of the present study also reinforce the modular theory of headache.[16] However, more studies of migraine and TTH subjects, using functional neuroimaging techniques, are required to clarify this issue.

This study had a few limitations. We used questionnaires for the present study and the reliability of this instrument has always been questioned, except in a few studies.[5,17–18] However, other studies suggest that it is a reliable and valid measure of sleep timing (including time to bed, wake-up time, total sleep time and sleep latency) and correlates well with traditionally used sleep diaries[19–20] and the actigraphy, specially for school nights rather than corresponding weekend nights.[20] As far as the diagnosis of headache is concerned, Laurell *et al.*[21] reported that the questionnaires are a valid source of information and that diagnosis of headache may be based upon them, except the cases of low intensity headache. Also, the short semi-structured interviews based on typical headache characters are an efficient and reliable method for the diagnosis of migraine and tension type headache among adolescents.[22] However, we opine that population based prospective studies with the objective evidence of sleep parameters must be planned in future. Such studies, when conducted in specialty clinic based population, suffer from selection bias, as most of the patients with headache do not ask for medical help unless it becomes really troublesome.

In essence, this study has demonstrated that sleep parameters are affected by some internal factor among migraineurs, and to a lesser extent in TTH sufferers. It is an evidence of possible neurobiological link between migraine and sleep disorders. It underscores the need for sleep assessment of migraineurs and TTH patients.
